# Supplementary material for: Phenotype of White Sika Deer Due to SCF Gene Structural Variation
Source: Genes (Basel). 2023 May 2;14(5):1035. doi: 10.3390/genes14051035 (PMC10218528; doi:10.3390/genes14051035)
Supplement: Supplementary file 1 [file genes-14-01035-s001.zip › genes-2250703-supplementary.pdf]

Table S1 List of candidate genomes

Gene	function
KIT	Mast/stem_cell_growth_factor_receptor_Kit
CORIN	Atrial_natriuretic_peptide-converting_enzyme
AP3B1	AP-3_complex_subunit_beta-1
HPS6	Hermansky-Pudlak_syndrome_6_protein_homolog
HPS1	Hermansky-Pudlak_syndrome_1_protein
MC1R	Melanocyte-stimulating_hormone_receptor
TYRP2	L-dopachrome_tautomerase
EDNRB	Endothelin_B_receptor
HPS4	Hermansky-Pudlak_syndrome_4_protein
HPS4	Hermansky-Pudlak_syndrome_4_protein
AP3D1	AP-3_complex_subunit_delta-1
TYR	Tyrosinase
HPS5	Hermansky-Pudlak_syndrome_5_protein
CDC42	Cell_division_control_protein_42_homolog
SOX5	Transcription_factor_SOX-5
WRN	Werner_syndrome_ATP-dependent_helicase
DKK3	Dickkopf-related_protein_3
COPB	Coatomer_subunit_beta
TIMP3	Metalloproteinase_inhibitor_3
PTPRT	Receptor-type_tyrosine-protein_phosphatase_T
ASIP	Agouti-signaling_protein
ALX3	Homeobox_protein_aristaless-like_3
PMEL	Melanocyte_protein_PMEL
VDR	Vitamin_D3_receptor
MITF	Microphthalmia-associated_transcription_factor
HPS3	Hermansky-Pudlak_syndrome_3_protein
TRPM1	Transient_receptor_potential_cation_channel_subfamily_M_member_1
GGT1	Gamma-glutamyltranspeptidase_1
DSG4	Desmoglein-4
TRPM7	Transient_receptor_potential_cation_channel_subfamily_M_member_7
MYO5A	Unconventional_myosin-Va
IRF4	Interferon_regulatory_factor_4
EXOC2	Exocyst_complex_component_2
WNT3A	Protein_Wnt-3a
PTPRF	Receptor-type_tyrosine-protein_phosphatase_F
COPA	Coatomer_subunit_alpha
ATP7A	Copper-transporting_ATPase_1
EDN3	Endothelin-3
TYRP1	5,6-dihydroxyindole-2-carboxylic_acid_oxidase
PAX3	Paired_box_protein_Pax-3
MEF2C	Myocyte-specific_enhancer_factor_2C
AP3D1	AP-3 complex subunit delta-1

AP3B1	AP-3 complex subunit beta-1
AP3B2	AP-3 complex subunit beta-2
SLC45A2	Membrane-associated transporter protein
GPR143	G-protein coupled receptor 143
BLOC1S5	Biogenesis of lysosome-related organelles complex 1 subunit 5
BLOC1S6	Biogenesis of lysosome-related organelles complex 1 subunit 6
EPG5	Ectopic P granules protein 5 homolog
GNAI	Guanine nucleotide-binding protein G(i) subunit alpha-1
DTNBP1	Dysbindin
RAB27A	Ras-related protein Rab-27A
ADAMTS20	A disintegrin and metalloproteinase with thrombospondin motifs 20
HTT	Huntingtin
KDR	Vascular endothelial growth factor receptor 1
KITLG	Kit ligand
PDGFRA	Platelet-derived growth factor receptor alpha
CIITA	MHC class II transactivator
ARPC5L	Actin-related protein 2/3 complex subunit 5-like protein
POMC	Pro-opiomelanocortin (Fragment)
KRT2	Keratin, type II cytoskeletal 2 epidermal
CCT2	T-complex protein 1 subunit beta
CDC25A	M-phase inducer phosphatase 1
CREB3L2	Cyclic AMP-responsive element-binding protein 3-like protein 2
MYO10	Unconventional myosin-X
MYO6	Unconventional myosin-VI
MRAP/FALP	Melanocortin-2 receptor accessory protein
MREG	Melanoregulin
PIKFYVE	1-phosphatidylinositol 3-phosphate 5-kinase
POT1B	Protection of telomeres protein 1
VPS33A	Vacuolar protein sorting-associated protein 33A
ACVR2A	Activin receptor type-2A
DICKKOPF-3	Dickkopf-related protein 3
EDN1	Endothelin-1
EDN2	Endothelin-2
EDN3	Endothelin-3
CREB1	Cyclic AMP-responsive element-binding protein 1
CTNNB1	Catenin beta-1
ICAT	Beta-catenin-interacting protein 1
DKK1	Dickkopf-related protein 1
ATRN	Attractin
MGRN1	E3 ubiquitin-protein ligase MGRN1
GSK3beta	Glycogen synthase kinase-3 beta
Lef1	Lymphoid enhancer-binding factor 1
MLPH	Melanophilin
OCA2/P	P protein

SLC24A5	Sodium/potassium/calcium exchanger 5
SLC38A8	Putative sodium-coupled neutral amino acid transporter 8
LYST	Lysosomal-trafficking regulator
BLOC1S3	Biogenesis of lysosome-related organelles complex 1 subunit 3
SCF	Kit ligand
EDNRB	Endothelin B receptor

Table S2 Primer List

Gene	Primer	Length(bp)	Temperature of anneal
SCF-1	F: AGCGAACCACAGAGGACATA<br>R:TTTACTGAGAGGGGGCATTA	294	52℃
SCF-2	F:TACAAAAGCGAACCACAGAGG<br>R:TGGAAACCCAAAATAACATCA	1184	52℃
SCF-3	F: ATAACTCCCATACAAGACGG<br>R:GCAGAGACCCGTGGATGG	423	52℃
SCF-4	F:GTGGGAAAGACAACAGAGTAAT<br>R:GACATTGGTAGAGCCAGCATTT	326	55.1℃

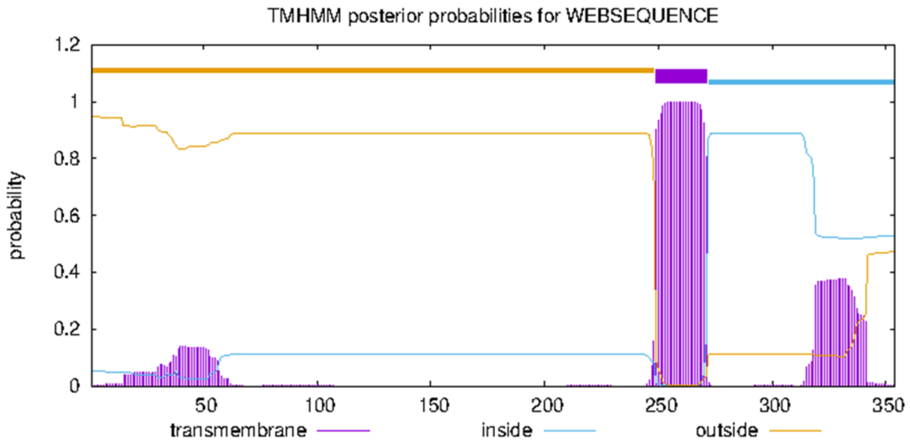

Figure S1 Structure prediction of SCF protein in sika deer outside, 1-248; Tmhelix, 249-271; inside, 272-354.
